# Glasgow Royal Infirmary
*Glasgow Med. Journ. No. V.


**Published:** 1829-07-01

**Authors:** 


### GLASGOW ROYAL INFIRMARY.*

### I. STRANGULATED HERNIA.

In no part of surgery have greater practical improvements taken place within the last half century than in hernia. The anatomical descriptions of the older authors are obscure, and frequently erroneous, and the bare perusal of the treatise of Mr. Pott upon ruptures, must shew satisfactorily how much we have added to our knowledge of the disease, and our modes of treating its different varieties, since the time of that able surgeon. Much, however, is yet a matter of dispute and doubt, which can only be removed, if they ever are to be removed, by carefully noting facts as they arise, and erecting on them, and them only, rigid and right conclusions. With this object in view, we are led to notice some cases of hernia, and tumour bearing the resemblance of hernia, that occurred to, and are detailed by Dr. Maclachlan.

*Case* 1.—*Omental and Intestinal Femoral Hernia—severe after Symptoms.* A woman, æt. 36, had been subject, for ten years, to reducible femoral hernia on the right side, and, for three, to the same on the left. The latter, however, descended in the course of the 15th September, 1827, and could not be returned by the patient as before—in the course of an hour the tumour became painful—retching and vomiting—quickly followed with extension of the pain to the abdomen—she was bled, and the taxis ineffectually employed for a quarter of an hour by a surgeon—and, in 44 hours after the first occurrence of the descent, th[illegible] patient was received in the Royal Infirmary. The tumour was then, 5 p. m. of the 1[illegible], about the size of a large hen's egg, elastic, and very tender

---

* Glasgow Med. Journ. No. V.

on being handled; there was much pain of abdomen, increased upon pressure; the pulse was 104, small, but firm; she vomited whatever she swallowed, and occasionally hiccuped; and, lastly, there had been no stool since the hernia descended. The warm bath, bleeding, and taxis were tried, without effect, and at 7 p. m. the operation was performed; the stricture, which was firm, divided by means of Cooper's knife; the contents of the sac, a knuckle of small intestine, and a little omentum, returned into the abdomen; and the patient was ordered ℥ss. of castor oil, and afterwards a laxative enema. The gut was of the colour of Port wine.

Numerous stools were had on the 18th, but some pain in the abdomen remained, and was aggravated next morning, when eighteen leeches were applied, but without much relief. Castor oil and an enema were ordered, and the former repeated twice, which brought away, upon the 20th, some dark and lumpy stools. The pain had increased so much during the night, that 16 ozs. of blood were abstracted with relief, though not perfect, to the symptom in question. On the 20th, the countenance had a tinge of yellow—the pulse was 120, rather full—tongue white—thirst moderate. Twelve leeches were applied to the abdomen, and a scruple of calomel exhibited. Aided by an enema, the calomel produced some dark-coloured stools, the pain of the abdomen subsided, the unfavourable symptoms passed away, and the patient was dismissed on the 9th of October.

"From the severity of the after symptoms, it will be evident, that any farther delay in having recourse to the operation must have proved fatal. It appears to me, that the active employment of the taxis should be confined to the earlier stages of the incarceration, while the contents of the sac are as yet but slightly inflamed. When, however, the tumour becomes extremely painful, and the constitutional symptoms indicate the supervention of high inflammatory action, the taxis will prove worse than useless. The intestine and omentum are now gorged with blood, and if any portion of mesentery has descended, it will be thickened from infiltration; the obstacles thus opposed to reduction will be greatly increased, while the chance of doing mischief is almost certain. In the following case, the frequent and unskilful employment of the taxis appears to have had no inconsiderable share in the production of the fatal result."

The particulars of the case alluded to are these.

*Case* 2.—A sailor, 46 years of age, had had a scrotal hernia of the right side, which was easily reduced and kept up by a truss. On the 26th of May, it came down, in consequence of neglecting the truss, his efforts were unable to return it, the usual symptoms of strangulation followed, and being at sea, without any medical aid at hand, his companions made numerous but unsuccessful efforts at reduction. At 5, p.m. of the 28th, when he entered the infirmary, a tumour appeared in the right side of the scrotum, very tense, and painful when handled, the integuments over it glossy and of dark red colour—pulse 100, small and wiry—tongue furred—great thirst—constant nausea and retching—skin hot and dry—face flushed—no stool for six and fifty hours.

"A consultation was immediately held, and the operation decided upon, without making any trial of the taxis. On opening the sac, about 15 inches of the ileum with its mesentery, were found strangulated. The intestine was of a deep red colour, but had not lost its natural shining appearance. The mesentery was firm, hard, and uncommonly thickened, partly from effused lymph, but mostly from ecchymosis. The thickness may be stated at ¼ of an inch. The obstacle to the return of such a mass into the abdomen, must be at once apparent. On passing the finger to the point of constriction, it was found so open, that it passed with ease into the cavity of the abdomen. The hernia being one of long standing, the upper and lower rings had, as it were, become one. Cooper's knife was introduced on the finger, and the edge of the transversalis muscle divided directly upwards, but it was only after repeated enlargements that this unyielding mass could be returned. This being accomplished, the wound was brought together, and dressed in the usual manner. He had castor oil with laudanum, and a laxative enema."

Several stools were obtained after the operation, but he passed a bad night; the peritoneal symptoms made head, notwithstanding the employment of two 16-oz. bleedings, and a large dose of calomel, depression supervened, and at 7, p.m. of the 30th, the patient sank.

*Dissection.* Marks of acute peritoneal inflammation were present, and the mesentery of the ileum, for about twenty inches, was from ¼ to ¾ of an inch in thickness, as well as firm and hard from effused blood. Three livid patches, the largest the size of a shilling, were found on that portion of gut which had been strangulated, although it had in no point actually given way. The constriction was formed "by the border of the transversalis muscle." The ring was much dilated, and admitted two fingers with ease—the spermatic cord was greatly enlarged.

In the following remarks on the use of the taxis we fully concur, and certain we are, that we have witnessed cases illustrating their truth.

"It will be evident from the size of the aperture by which the intestine had descended, that, had the taxis been scientifically employed previous to the infiltration of the mesentery, the contents of the tumour might have been readily returned: but it will be equally obvious, that, at any period subsequent to this event, it would have proved not only unavailing, but highly injurious. The ecchymoses arose, no doubt, from the rough efforts made by the sailors to reduce the rupture; but had the engorgement arisen simply from constriction, the same objection to the use of taxis would apply. I am no enemy to the employment of taxis; I only object to its indiscriminate adoption, without any reference to the history or stage of the case. I also believe that in many cases the fatal termination usually ascribed to the operation for strangulated hernia, might, with more propriety, be given to the repeated and long continued use of the taxis. In the two cases above related, the incarceration was nearly of the same duration. In the one, the hernia was smaller, more acute, and therefore more dangerous, the taxis was but little employed, the operation was had recourse to, and the patient recovered. In the other, the hernia was of long standing, the opening into the abdomen large, therefore comparatively less dangerous, many efforts were made, the operation was performed, and the patient died."

### II. HYDROCELE OF THE SPERMATIC CORD COMPLICATED WITH PERITONITIS.

It has long been known to surgeons, that encysted hydrocele of the spermatic cord, especially if situate in the inguinal canal, so as to receive the impulse of coughing, is extremely likely to be confounded with an inguinal hernia; and, indeed, under certain combinations of circumstances, the diagnosis, we conceive, must be next to impossible. Surgeons, we say, have long been aware of the occasional occurrence of this difficulty; but only in the present age have wretches been found so anti-professional and base, as to make it a theme of calumny and vituperation against individual practitioners of surgery. For an error of this kind, at St. George's Hospital, Mr. Jeffreys was "shewn up," (we believe that that is the phrase) and the one we are going to relate formed one of those tales of "butchery and blood" which graced the pages of the Lancet, from the pen of that very promising young gentleman, Mr. Carter.

*Case.*—James Porter, two years of age, was admitted, on the 24th of June last, with an oblong elastic tumour in the upper part of the right side of the scrotum. It was painful on being handled, the size of a small walnut, and entered and distended the external ring—the integuments of the scrotum moved freely over it, and were not discoloured—the cord felt enlarged, and lay immediately below and posterior to the tumour, but separated from it—the cord was behind it and felt enlarged—the abdomen was distended, tympanitic, and the child cried when it was touched, particularly below—there had been, for eight days, no motion from the bowels; and nausea and retching for the last two—the pulse was 120—skin hot—tongue white—the thirst extreme. His mother said that she first observed this tumour, three months before, in the situation of the external ring; that it gradually enlarged, and decended towards the scrotum—that pressure made it disappear, which it did with a gurgling noise; that she had been *recommended by a surgeon to get a truss to keep it up;* that it had continued in the situation it was in on his admission for two weeks before that time; and that, in consequence of the constipation and vomiting alluded to above, a surgeon had been called in, who had tried, without effect, to reduce it by the taxis.

"Here, then, were all the symptoms of incarcerated bowel, with a tumour filling the external ring, which, from its situation, and the smallness of its size, could not, by any possibility, be distinguished from inguinal hernia. The taxis was tried, but without any change in the tumour. As the symptoms, as yet, were not very urgent, and as the incarceration appeared to be rather *par engouement* than strangulation, the boy was ordered (at 2 P.M.) 3 grains calomel; in an hour after to have ʒij. castor oil, and in two hours more, a laxative enema, and to be placed in the warm bath. Should these means afford no relief, a consultation to be summoned for 6 P.M.

"The enema brought away a pretty large stool, composed entirely of hardened balls and some blood; but there was no indication of the medicines given by the mouth having contributed in any way to it.

"Consultation met at six o'clock. No relief from the stool. The boy was more feverish and restless. Complained more on the tumour being handled. Abdomen still swollen, hot, and tender to touch. Pulse 140. Skin parched. Face flushed; in short, a considerable aggravation of all his symptoms. A moderate trial of the taxis having again failed, it was decided to open the sac, and ascertain the nature of its contents. At 7 P.M, this was done in the usual manner, and on laying open the investing membrane, a gush of clear fluid, followed by a considerable quantity of thick jelly, was pressed out, showing the tumour to be hydrocele of the cord."

The parts were brought together, and a compress lightly applied, whilst three grains of calomel, and afterwards infusion of senna, were given to procure evacuations from the bowels, which took place on the following morning, although, at that time, he was still very feverish, and cried when the belly was touched. We need not follow out the details of the case, suffice it to say, that although a temporary improvement took place, the child fell back again, got gradually lower, and died on the 4th of July, just ten days after his admission.

On dissection, the abdomen contained nearly a pint of purulent fluid, the peritoneum was pale, the intestines were enormously distended with flatus, and at some points slightly adherent. The spermatic cord, from the testis to the inner ring, was hard, and enlarged to nearly three times its natural size, whilst, within its sheath, an abscess was found, containing two drachms of pus. The left side of the chest contained about twelve ounces of sero-purulent fluid, and the lung was covered with a thick layer of lymph.

"All surgical writers agree as to the extreme difficulty of distinguishing hydrocele of the cord, when small, and when it distends the external ring, from inguinal hernia, even when none of the symptoms of strangulated bowel are present; but when peritonitic symptoms exist, with absence of stools, discrimination becomes absolutely impossible. Even Mr. Pott, a competent judge, one would think, was much puzzled in a case of hydrocele of the cord, where *no* febrile symptoms were present. A lad of 14 had a large tumour, full and tight, possessing the whole spermatic process and scrotum down to the testicle, which was independent of it, not tender to touch, unless pressed hard. It bore so hard against the opening of abdominal muscles, that Mr. Pott could by no means feel the spermatic process. He had had no stool for five days. 'Some of these circumstances were of importance, and

N 2

might be occasioned by a stricture on the intestinal canal; but, on the other hand, his pulse was soft, calm, and quiet. His skin was cool; he had neither tight belly, nausea, hiccough, nor vomiting, nor any other symptom deducible from such a cause. From the mere appearance and feel of the tumour, I should have supposed it to be caused by water; but the difficulty of distinguishing the spermatic process above, the freedom of the testicle below, and the want of stools, made me hesitate.' He tried the taxis, and not succeeding, he, in the absence of urgent symptoms, gave him a purging mixture and an enema, and bled him. His medicine operated in *two hours*, and he was relieved. But Mr. Pott was not satisfied. 'I examined it again and again, and was still more positive that it contained a fluid; but whether that fluid was in the tunica vaginalis, or in a hernial sac, I could by no means be clear. However, as there was no possible means of getting rid of it but by an opening, I determined to make one with such caution as to be prepared for whatever might happen.' The treatment adopted in both cases was precisely the same, only in Mr. Pott's case bleeding was had recourse to; but surely the diagnosis was much more difficult in my case than in Mr. Pott's. Other cases might be cited to prove the difficulty of diagnosis. I shall content myself, however, with the following from Mr. Dewar's paper in the Edin. Med and Surg. Journal. 'I was requested by Dr. Stenhouse to visit a patient of his, who was labouring under hernia. The man was suffering from pain and distention of the belly; frequent vomiting, and obstinate costiveness. There was a firm elastic swelling, about the size of a pigeon's egg, occupying the inguinal canal. He complained of very little pain on handling the tumour. When an attempt was made to return it into the belly, it receded in a slight degree within the canal; but returned to its former situation immediately on the fingers being removed. All the usual means of effecting reduction were tried without success, and the operation determined upon. When the tumour was opened by Dr. Stenhouse, a small quantity of a glairy fluid ran out, and the finger was passed to the second joint, in a shut sac, which had no communication with the abdomen. Of course, no relief followed. His bowels, however, ultimately yielded, and he got well. I do not yet know, after frequent reflection upon this very interesting case, how it could be distinguished with certainty from hernia.'

"The circumstance of a stool being passed is by no means decisive of the absence of strangulation. An enema frequently brings off fæces from the colon and rectum, in cases of incarceration. Mr. Tyrrell mentions a case in which even four copious stools were passed, notwithstanding the existence of strangulation, which afterwards required the operation. 'The sac contained a portion of intestine highly inflamed, and perfectly incarcerated.' The man died, and, on dissection, the following is stated as the condition of the gut:—'The portion of intestine which had been strangulated consisted of a complete fold of the ilium, including the whole diameter of the gut; it had still the mark from the stricture upon it, and was much more discoloured than any other part.' The fæces, therefore, could not have passed this constriction; they must have been furnished by the bowel lower down than the point of strangulation. It is evident, from the case above detailed, that the child laboured under peritonitic, and perhaps pleuritic symptoms on admission, the consequence of long-continued constipation, and the irritation of worms—That there existed a tumour in the situation of inguinal hernia, which could not be distinguished from that disease—That the history furnished by the mother was every way calculated to mislead—That there was constipation of many days' continuance. The taxis having failed, and the inflammatory symptoms continuing to increase, there was no alternative left for the surgeon. He must have ascertained the nature of the contents of the sac: if hernia, he was right; if hydrocele of the cord, he could scarcely be said to be wrong, for this also requires such an operation for its cure."

When Dr. Machlachlan remarks, that all surgical writers are agreed on the extreme difficulty of distinguishing hydrocele of the cord from hernia, he is wrong, most decidedly wrong. It is true that Mr. Pott was somewhat puzzled, that Sir Astley Cooper sees a difficulty, that Mr. Jefferys and the surgeons of the Glasgow Infirmary, and Dr. Stenhouse, and many other people have at different times been mistaken, but what, we would ask, are Mr. Pott and Sir Astley, and the rest, to the able, the distinguished, the practical writers in the Lancet? *They* find no difficulties—*they* make no blunders—*they*, and they alone, are exempt from uncertainty and doubt. What! though the hospital reporter for the Lancet be a mere raw student, who never fledged a blade, save his own peculiar weapon, on any human being in his life—what, though if put to treat cases himself, he probably could not tell an aneurism from a rupture—what, though the natural and ancient order of things be reversed, and the pupil be the critic of his master, not the master the instructor of the pupil; yet think of the liberty of the press; think of the sacred office of the Lancet; think of its imperious obligation to revile the profession, and every respectable man to be found in it! Aye, your men "of warm feelings," are the only men that are really "independent,"—and fit to be entrusted with the characters of their neighbours. Vive la calomnie!

### GLASGOW ROYAL INFIRMARY.*

### I. NÆVUS MATERNUS CURED BY VACCINATION.

We have no great faith in the powers of vaccination over nævus maternus; in fact its occasional success is one of those pretty little facts which are rather a matter of curiosity than of practical im-

---

* Glasgow Journal, No. VI.

portance; although it is well to be aware that in some few cases, one perhaps in fifty, it will save the employment of severer measures. A child, for instance, eight months old, was brought to Dr. Auchincloss, at the Glasgow Infirmary, last September, with a nævus on the forehead, as large as a hazel nut, and of dark red colour. It was present at birth, but attained no elevation from the surface until after a month. The child not having been vaccinated, fresh lymph was inserted by minute punctures, both around the circumference, and over the whole extent of the tumour. On the 8th day, many small pustules were visible; on the 12th, they had coalesced and become incrusted; on the 21st, the scab separated; a second crust succeeded, then a third, and a fourth; and in six weeks a perfect cure was obtained.

II. LUMBAR ABSCESS POINTING BEHIND THE TROCHANTER.

The situations in which lumbar abscess points we have reason to believe are much more numerous than is commonly imagined. In one or two instances we have seen it present above the trochanter major and below the anterior portion of the crest of the ileum. In the case we are now to relate, it took another, and yet not very dissimilar direction. The patient, a delicate lad of 14, had laboured under constant pain in the back for eleven months, to which had been added, "diarrhœa and *gripes*," (not a very elegant mode of expressing it) for the last six weeks, when he was received in the infirmary. At this time there was a painful fluctuating swelling over the right hip from the trochanter backwards, which had not been present above three weeks. Under the employment of opiates with chalk mixture, and a succession of blisters to the back and hip, the diarrhœa passed away, and the pain and swelling also subsided. The bowel complaint, however, returned, with tenesmus and tormina; the swelling behind the trochanter suddenly disappeared; hectic was established, and the patient sank.

On dissection it was found that a large thickened cyst, half filled with pus, extended from the lower part of the sacrum to the upper edge of the last lumbar vertebra. From its middle part a sinus issued, left the pelvis through the ischiatic notch, and led to a large empty sac behind the trochanter. The "cyst" on the sacrum also communicated anteriorly with the rectum by a fistulous opening the size of a crow-quill. "The mucous coat of the rectum and lower part of the ilium" was of a deep red colour and covered with numerous minute ulcers. The inferior lumbar vertebra and upper portion of the sacrum were carious, and their inter-vertebral cartilage destroyed.

This dissection is of value, as all such dissections undoubtedly are, by assisting diagnosis in a similar case. By the bye, we suspect that an error has crept into Dr. Auchincloss's notes of the case, for it seems somewhat odd that the *rectum* and lower extremity of the *ileum*, parts so remote from each other, should present precisely the same morbid changes. Was it the *cæcum* and lower portion of the ileum that were thus affected?

III. SLOUGHING OF THE SCROTUM.—CURIOUS SWELLING IN THE PERINEUM.

*Case* 1. A man, 37 years of age, had been under treatment in the medical wards for a bowel-complaint, when, at the end of three months he was dismissed cured. A week after his dismissal he presented himself again and was re-admitted for a hydrocele of the right side larger than the fist. The integuments over its middle and fore part, to the extent of half a crown-piece, were of pale red colour, œdematous, and painful. A bag-truss, spirit lotion, opiate enemata, and generous diet, were the means prescribed, but the swelling and redness gradually extended, and, on the 2nd of September, four days after his admission, the whole scrotum was affected, and presented a swelling much larger than a child's head. During the preceding night a distinct tumour, the size of a hen's egg, had formed in the perinæum, occasioning retention of urine, and necessitating the introduction of the catheter, which was effected without the least difficulty. The gum catheter being in, an incision was made on the perinæal tumour, "down on the urethra," in the direction of the raphé, but only a trifling quantity of serum escaped. Several punctures were also made over the scrotum; a poultice was applied; and wine with opium and opiate enemata exhibited. Though the punctures and incision procured relief, the tumour in perinæo growing flaccid, and that in the scrotum being less, yet sloughing of the latter took place, typhoid symptoms ensued, and the patient expired on the 5th.

*Dissection.* The whole of the scrotum was in a state of sloughing. The cellular tissue of the perineum, as far back as the tuberosities of the ischium, was sloughy and infiltrated with pus—the urethra was pervious throughout, and admitted a full-sized sound—the inner coat of the rectum and lower part of the colon, was much thickened and studded with numerous ulcers.

"The preceding case excited considerable interest. On the occurrence of the perinæal tumour, on the 4th day, it bore a striking resemblance, in every particular, both constitutional and local, to a case of urinary abscess. The gentlemen, to whom allusion has been made, did not see the patient till the above advanced period, when judging principally from the appearance which the swelling presented, without adverting to the previous history, they were more readily misled in their opinion concerning it. Having witnessed the progress of the case from the commencement, I felt confident that it was in no way connected with ulceration of the urethra or bladder, for the following reasons: 1st, As no previous retention, or difficulty in voiding urine, had been experienced: 2dly, As a full-sized catheter could be easily introduced: and 3dly, The œdematous inflamed patch having first appeared on the middle and anterior part of the scrotum, midway between the septum and angle of the thigh. On the other hand, had it been the consequence of urinary infiltration, I should imagine the redness and swelling would have first shewn themselves either contiguously to the septum, at the root of the penis, or in the lower part of the groin. Besides, tension of the perinæum, in all probability, would have preceded these appearances.

"It is merely an instance of sloughing of the cellular substance supervening on erysipelas, in an unhealthy constitution, in connexion, I should suppose, with the diseased condition of the rectum and colon. Erysipelas, and other cutaneous affections, are frequently met with as symptomatic of visceral disease.

"Respecting the propriety of the practice by incision, there can be little doubt. It was had recourse to on somewhat the same principles as in cases of urinary abscess, namely, the relief of tension, evacuation of sloughy matter, and prevention of the gangrenous action."

*Case* 2. W. Laverty, a sickly-looking child, 18 months old, was admitted at noon of the 25th September, having voided no urine, since 5, p.m. of the preceding day. The penis and scrotum were swollen, the latter larger than a man's fist, hot, tense, shining, and translucent. There was no tension or fulness in the perineum, and but little in the hypogastrium; the child was feverish, and had suffered from bowel-complaint for the last three months. Twelve ounces of urine were readily drawn off by the gum elastic catheter which was left in the bladder; punctures were made in the swollen parts; and fomentations ordered. The little patient was relieved—in the evening the parts were much less swollen—and on the following day the swelling had almost disappeared. The urine now issuing freely by the side of the catheter, it was withdrawn, but next day the tumefaction had returned to nearly its former extent, accompanied with hardness and a dark red colour of the scrotum. The child was now taken home by its mother, sloughing took place on the lower part of the scrotum and root of the penis, the urethra was exposed, then opened by ulceration, and on the 9th of October the patient sank.

*Dissection.* "The kidneys were twice their natural size, and lobulated. The right was the larger, and the more diseased of the two. On section, several small encysted abscesses appeared in their substance, which was unusually soft, and of a pale gray colour. The ureters were dilated to three times the diameter of a common crow quill. The bladder was thickened, and very much contracted, and its inner surface of a dark red colour, and studded over with fungous tumours. These varied in size, from that of a pea to that of a marble; and appeared to be seated under the mucous coat. The larger ones were distinctly fibrous, while the smaller consisted chiefly of black substance, resembling small clots of blood, seated immediately below the inner coat. The prostate gland was much larger than natural, but its structure was free from disease. There was extensive ulceration of the inner surface of the ileum and colon."

As Dr. Auchincloss observes, it is certainly rare to meet with so much organic disease in a child not two years old. Dr Auchincloss attributes the sloughing of the penis and scrotum to the child's having frequently pulled at the prepuce, in consequence, no doubt, of irritation referred to the extremity of the penis, though depending on the disease of the bladder within. The prepuce, it seems, was the first part to swell, but still the idea that the destructive sloughing of the penis and scrotum resulted from the little mechanical cause in question, looks rather far-fetched, to say the least of it. The lengthened prepuce is almost a constant concomitant of calculus in the bladder of children, but we never saw any such direful sequelæ of the practice as sloughing of the neighbouring parts. These two not dissimilar cases are instructive examples of that dangerous erysipelatous inflammation to which the parts in the perinæum are prone—quite independently of urinous extravasation. We cannot recommend too strongly in both the practice of incisions, a practice which, if not attended with uniform success, is so in the majority of cases when timely and boldly performed.

### IV. Partial Palsy cured by Strychnia locally applied.

An habitual drunkard, aged 56, was admitted into the infirmary, having suddenly lost the power of the left fore-arm and hand ten days previously. The sensation of the parts was perfect, but the power was much impaired—he had no head-ache—the bowels were costive after free purging—a blister was applied to the back of the fore-arm, and $\frac{1}{8}$th of a grain of strychnia sprinkled over the vesicated surface. On each successive day the application was increased by adding the original quantity to that of the preceding day, till it amounted to one grain, after which $\frac{1}{4}$th of a grain, instead of $\frac{1}{8}$th, was to be added. From the second week the improvement was manifest, no obvious constitutional effect ensued, and the patient was dismissed cured at the end of five weeks from the commencement of the treatment. In another patient affected with paralysis of the flexor muscles, and diminished sensation of the right leg, a cure was effected in the course of six weeks.

### V. Affection of the Legs resembling Elephantiasis.

A man at the age of 60 suffered compound fracture of both bones of the right leg, followed by inflammation and sloughing that protracted the cure for the space of five months. Shortly after this the legs began to swell; the swelling gradually extended upwards to the trunk; openings then formed in the scrotum, thighs, and legs, and gave issue to an immense discharge of watery fluid, in consequence of which the enlargement grew daily less, and at length the openings healed. Both legs still continued a little swollen, and nine months before his admission the right one began again to enlarge and to grow more firm, whilst ulcerated chaps broke out upon its surface. The left leg shewed no increase for several months after this, when it ulcerated like the right. The urine was rather scanty, and he had been subject to dyspnœa for several years, but otherwise his health was good. On the 28th July, 18 months after the accident, he was admitted into the infirmary, with the right leg and foot more than treble their natural size; the swelling uniformly firm and resisting, though pressure produced a trifling pitting; the skin much thickened, and elevated into numerous small nodules along the fore-part of the leg and dorsum of the foot, but rough and covered with a scurf behind; in the interstices of the nodules superficial ulceration discharging a serous offensive fluid, forming brownish incrustations on exposure; the swelling of the leg and that of the foot separated in front by a fissure extending across the ankle-joint an inch and a half in depth, and sufficiently broad to permit the finger being passed to its bottom. The left leg was swollen to nearly double its natural size, whilst the integuments on its lower half were ulcerated, and presented the same tuberculated appearance as those on the opposite limb.

Such were the history and symptoms presented by the patient, and the following the treatment had recourse to. On the 8th, he was ordered two grains of calomel, one of squill, and one of ginger, thrice daily, and applied locally a piece of caddis moistened with a lotion of two ounces of liquor potassæ to a pint and a half of water, whilst the limbs were likewise firmly bandaged. The ulceration under this treatment soon dried up, the legs sensibly diminished in thickness, and on Sept. 4th, the surface of both had lost its fissured appearance. He generally voided 5lbs. of urine daily, under the use of the powders, which at the last-mentioned date were omitted in consequence of the mouth being affected, but the topical means were persisted in, and on the 13th of October the patient left the house. The left leg was at this time of its natural thickness; the right still continued a little enlarged, although the swelling was perceptibly subsiding.

"The preceding case appears to me rather anomalous. I am altogether in doubt by what name it ought to be designated. So far as appearances were concerned, it certainly answered the description given of elephantiasis better than of any other disease which I have either seen or read of. Nevertheless, from the history of the case, and the result of the treatment, it may, perhaps, be supposed to have been merely a chronic œdematous affection. I have to remark, however, that it was not recognised as being of this character by any of my medical friends who saw the patient a short time after his admission. The impression left after firm pressure with the point of the finger was exceedingly trifling; which may be easily explained, I apprehend, by the circumstance, that poultices had been applied to both limbs for a fortnight previously.

"Elephantiasis is a disease which originates in the subcutaneous cellular substance, and may, therefore, be said to consist in true hypertrophy of its structure. As the disease advances, this tissue acquires great increase of volume and density, ultimately affecting the skin, which becomes thickened, rugous, and elevated into tubercles. Several years ago, I had an opportunity of witnessing the examination of a leg affected with this disease, which was amputated by Dr. Robertson in the Royal Infirmary. The cellular structure was 6½ inches, and the skin upwards of 1 inch in thickness. The leg itself measured nearly four feet in circumference. On making longitudinal sections down to the fascia, about a gallon and a half of serum drained off. The skin and contiguous cellular substance, to the depth of half an inch, were exceedingly dense. The latter became softer in texture as it was more deeply seated, and close upon the fascia the cells were greatly enlarged, and contained serum, mixed with a glairy matter, something like white of egg. The fascia was thickened, but the muscles underneath were healthy, though somewhat pale. The disease had been of sixteen years' duration, and began after repeated attacks of erysipelas."

That this was an affection resembling in a degree, and that not a very remote one, the elephantiasis, we are perfectly willing to admit; but we cannot go so far as Dr. Auchincloss has gone, nor consider the resemblance as actual identity. The scanty urine, dyspnœa, slight pitting upon pressure, and history of the disease, are closer traits of anasarcous or œdematous affection than Dr. Auchincloss would seem to think, and the rapid improvement under the exhibition of calomel and squills, with local pressure, are no mean items in the features of the case by which our judgment should be guided. The right leg was that in which this curious affection, whatever it may be, had run to the greatest height, and this, too, was the limb that had suffered from compound fracture. Now every one must know how commonly œdematous affections are left as the sequelæ of fractures in a limb, and also how frequently these chronic infiltrations or affections of the subcutaneous cellular membrane will be followed by morbid action in the skin. We by no means say that the singular disease in the present case, was simply the result of the local injury, but we cannot allow, from several considerations, that it was absolutely the same as elephantiasis, nor indeed so like it as the able narrator has considered it.

One word before we part on the subject of amputations. Amputations, we are told, are all performed by transfixion with Lisfranc's knife, and this, says Dr. A. "is certainly a much neater, more speedy, and less painful mode of operation than by the double circular incision." We know not how it may be at Glasgow, but we know well that the highest surgical authorities here have come to just the opposite conclusion. In the majority of cases, the general opinion "certainly" is, that the flap operation deserves scarcely one of the commendations here awarded it.

